# Accuracy of detecting residual disease after neoadjuvant chemoradiotherapy for esophageal squamous cell carcinoma (preSINO trial): a prospective multicenter diagnostic cohort study

**DOI:** 10.1186/s12885-020-6669-y

**Published:** 2020-03-06

**Authors:** Xiaobin Zhang, Ben M. Eyck, Yang Yang, Jun Liu, Yin-Kai Chao, Ming-Mo Hou, Tsung-Min Hung, Qingsong Pang, Zhen-Tao Yu, Hongjing Jiang, Simon Law, Ian Wong, Ka-On Lam, Berend J. van der Wilk, Ate van der Gaast, Manon C. W. Spaander, Roelf Valkema, Sjoerd M. Lagarde, Bas P. L. Wijnhoven, J. Jan B. van Lanschot, Zhigang Li

**Affiliations:** 1 Department of Thoracic Surgery, Shanghai Chest Hospital, Shanghai Jiao Tong University, 241 West Huaihai Road, Shanghai, 200030 China; 2 Department of Surgery, Erasmus MC – University Medical Center, Dr. Molewaterplein 40, Rotterdam, the Netherlands; 3 Department of Medical and Radiation Oncology, Shanghai Chest Hospital, Shanghai Jiao Tong University, 241 West Huaihai Road, Shanghai, China; 4 Department of Thoracic and Cardiovascular Surgery, Chang Gung Memorial Hospital-Linkou Medical Center and Chang Gung University, 5 Fu-Shin Street, Kwei-Shan, Taoyuan, Taiwan; 5 Department of Hematology / Oncology, Chang Gung Memorial Hospital-Linkou Medical Center and Chang Gung University, 5 Fu-Shin Street, Kwei-Shan, Taoyuan, Taiwan; 6 Department of Radiation Oncology, Chang Gung Memorial Hospital-Linkou Medical Center and Chang Gung University, 5 Fu-Shin Street, Kwei-Shan, Taoyuan, Taiwan; 7 Department of Radiation Oncology, Tianjin Medical University Cancer Institute and Hospital / National Clinical Research Center for Cancer, West Huanhu Road, Hexi District, Tianjin, China; 8 Department of Esophageal Cancer, Tianjin Medical University Cancer Institute and Hospital / National Clinical Research Center for Cancer, West Huanhu Road, Hexi District, Tianjin, China; 9 Department of Surgery, Queen Mary Hospital, The University of Hong Kong, 102 Pokfulam Road, Hong Kong, China; 10 Department of Clinical Oncology, Queen Mary Hospital, The University of Hong Kong, 102 Pokfulam Road, Hong Kong, China; 11 Department of Medical Oncology, Erasmus MC – University Medical Center, Dr. Molewaterplein 40, Rotterdam, the Netherlands; 12 Department of Gastroenterology and Hepatology, Erasmus MC – University Medical Center, Dr. Molewaterplein 40, Rotterdam, the Netherlands; 13 Department of Nuclear Medicine, Erasmus MC – University Medical Center, Dr. Molewaterplein 40, Rotterdam, the Netherlands

**Keywords:** Esophageal cancer, Squamous cell carcinoma, Neoadjuvant chemoradiotherapy, Response, Residual disease, Accuracy, Sensitivity, Esophagectomy, Organ-sparing, Active surveillance

## Abstract

**Background:**

After neoadjuvant chemoradiotherapy (nCRT) for esophageal cancer, high pathologically complete response (pCR) rates are being achieved especially in patients with squamous cell carcinoma (SCC). An active surveillance strategy has been proposed for SCC patients with clinically complete response (cCR) after nCRT. To justify omitting surgical resection, patients with residual disease should be accurately identified. The aim of this study is to assess the accuracy of response evaluations after nCRT based on the preSANO trial, including positron emission tomography with computed tomography (PET-CT), endoscopy with bite-on-bite biopsies and endoscopic ultrasonography (EUS) with fine-needle aspiration (FNA) in patients with potentially curable esophageal SCC.

**Methods:**

Operable esophageal SCC patients who are planned to undergo nCRT according to the CROSS regimen and are planned to undergo surgery will be recruited from four Asian centers. Four to 6 weeks after completion of nCRT, patients will undergo a first clinical response evaluation (CRE-1) consisting of endoscopy with bite-on-bite biopsies. In patients without histological evidence of residual tumor (i.e. without positive biopsies), surgery will be postponed another 6 weeks. A second clinical response evaluation (CRE-2) will be performed 10–12 weeks after completion of nCRT, consisting of PET-CT, endoscopy with bite-on-bite biopsies and EUS with FNA. Immediately after CRE-2 all patients without evidence of distant metastases will undergo esophagectomy. Results of CRE-1 and CRE-2 as well as results of the three single diagnostic modalities will be correlated to pathological response in the resection specimen (gold standard) for calculation of sensitivity, specificity, negative predictive value and positive predictive value.

**Discussion:**

If the current study shows that major locoregional residual disease (> 10% residual carcinoma or any residual nodal disease) can be accurately (i.e. with sensitivity of 80.5%) detected in patients with esophageal SCC, a prospective trial will be conducted comparing active surveillance with standard esophagectomy in patients with a clinically complete response after nCRT (SINO trial).

**Trial registration:**

The preSINO trial has been registered at ClinicalTrials.gov as NCT03937362 (May 3, 2019).

## Background

### Rationale

Esophageal cancer is the seventh most common cancer and the sixth most common cause of cancer-related death worldwide. Highest incidence rates are found in Asia, with over 90% of patients having squamous cell carcinoma [1]. Of all patients with esophageal cancer worldwide, roughly half live in China. Almost half of the patients present with locally advanced disease and can be curatively treated with neoadjuvant chemoradiotherapy (nCRT) followed by surgery [2]. The CROSS trial showed that after nCRT consisting of carboplatin and paclitaxel with concurrent 41.4 Gy radiotherapy, 23% of patients with adenocarcinoma (AC) and 49% of patients with squamous cell carcinoma (SCC) have a pathologically complete response (pCR) in the resection specimen [3, 4]. For these patients surgical resection might not be necessary. Hence, an active surveillance strategy has been proposed in which patients will undergo frequent clinical response evaluations instead of standard esophagectomy [5].

Active surveillance is currently being investigated in two European clinical trials, i.e. the Dutch SANO trial and the French ESOSTRATE trial, including patients with both AC and SCC [6, 7]. Since the highest rate of pCR after nCRT is achieved in patients with SCC, an active surveillance strategy is highly relevant for Asian patients as well. This provides a rationale for a clinical trial comparing active surveillance with standard esophagectomy in patients with SCC showing a clinically complete response after nCRT (Surgery If Needed for Oesophageal cancer (SINO) trial). However, to justify omitting a potentially curative surgical resection patients with residual disease should be accurately identified. The preSANO trial showed that a combination of fluorodeoxyglucose (18F-FDG) positron emission tomography plus computed tomography (PET-CT), esophagogastroduodenoscopy (EGD) with bite-on-bite biopsies and endoscopic ultrasonography (EUS) with fine-needle aspiration (FNA) can identify substantial residual disease (> 10% vital tumor cells, tumor regression grade (TRG) 3–4) with a sensitivity of 90% [8].

Since the majority of patients (78%) included in the preSANO trial had adenocarcinoma and only 21% had squamous cell carcinoma, it remains unclear whether residual squamous cell carcinoma after nCRT can be accurately identified.

### Aim

The aim of the present study is to assess the accuracy of response evaluations according to the preSANO trial outcome after nCRT according to the CROSS regimen in patients with esophageal squamous cell carcinoma.

## Methods

### Study design and recruitment

The preSINO trial is a prospective, multicenter, diagnostic cohort study. The study will be conducted in four high-volume Asian hospitals, i.e. Shanghai Chest Hospital (coordinating center), Taiwan Chang Gung Memorial Hospital, Tianjin Medical University Cancer Center and Queen Mary Hospital of Hong Kong. The study is planned to be initiated in August, 2019, and results are expected in August, 2022. The trial has been registered at ClinicalTrials.gov as NCT03937362.

### Eligibility criteria

Esophageal cancer patients who are planned to undergo nCRT according to the CROSS regimen and who will undergo surgical resection will be recruited for this study. Patients will be considered eligible according to the following criteria.

Inclusion criteria are:

1. Histologically confirmed esophageal squamous cell carcinoma;
2. Tumor located in the chest;
3. Clinical stage cT1N1–2 M0, cT2–4aN0-2 M0, according to the 8th Edition of the AJCC TNM classification for Esophageal Cancer, where regional lymph nodes with a diameter ≥ 10 mm and lymph nodes around the left and right recurrent laryngeal nerve with a diameter ≥ 6 mm on CT as well as lymph nodes with a focal FDG signal above the adjacent esophageal background uptake are considered positive;
4. Age > 20 at the date of informed consent;
5. Eastern Cooperative Oncology Group (ECOG) Performance Status of two or less;
6. Considered fit to undergo nCRT followed by surgical resection;
7. Expected survival time more than 3 months;
8. Written informed consent by the patient.

Exclusion criteria are:

1. Patient with a second primary tumor;
2. Previous major surgery in the chest or upper abdomen;
3. Tumor not 18F-FDG-avid at baseline PET-CT;
4. Suspected positive lymph nodes that cannot be covered by an uninterrupted radiation field that also includes the primary tumor area;
5. Primary (early) lesion already removed by EMR/ESD;
6. Previous history of chemotherapy and/or radiation therapy;
7. Cervical esophageal cancer.

### Study process (Fig. 1 and Table 1)

#### Baseline examination and inclusion

At baseline, patients will undergo an esophagogastroduodenoscopy (EGD) with conventional tumor biopsy, endoscopic ultrasonography (EUS) in case of a traversable tumor, external ultrasound of the neck, high resolution CT of the neck, chest, abdomen and pelvis and whole-body PET-CT to stage the tumor and to exclude distant dissemination (Table 1). Preferably, photographs will be taken during EGD for future reference. Cytology or histology must be obtained from any suspected lymph nodes outside the planned radiation field. Moreover, quality-of-life questionnaires will be obtained. Written informed consent for study participation will be obtained from eligible patients after baseline diagnosis and staging (Table 1).

**Fig. 1:**
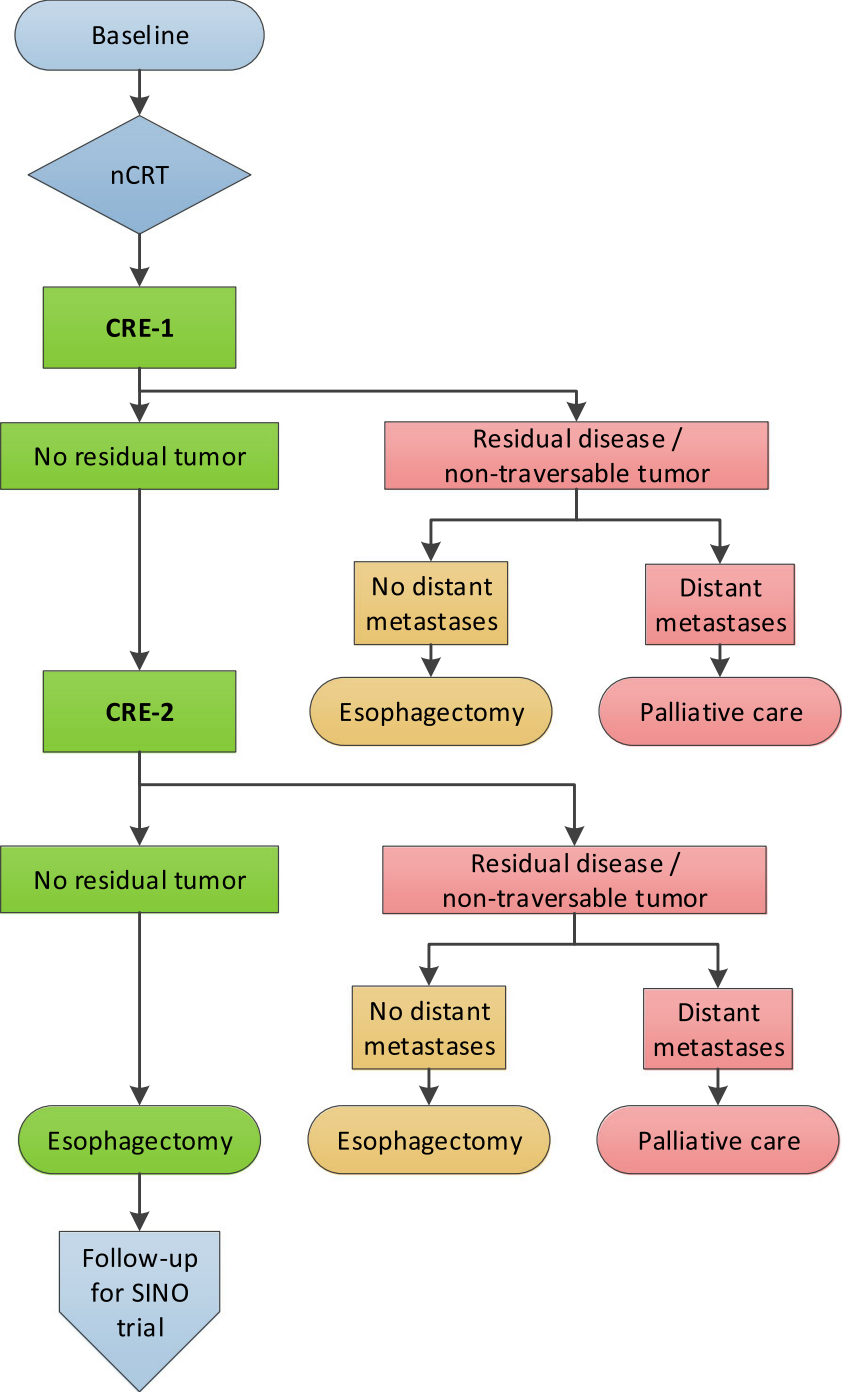
Flow chart of the preSINO trial. nCRT: neoadjuvant chemoradiotherapy; CRE-1: first clinical response evaluation, four to six weeks after completion of nCRT; CRE-2: second clinical response evaluation, 10–12 weeks after completion of nCRT

**Table 1:** Study process

Parameter	Baseline	nCRT	CRE-1	CRE-2	Surgery	Follow-up
History, physical examination	Yes	Yes	Yes	Yes	Yes	Yes
ECOG Performance Status	Yes	Yes				
Hematology^a^	Yes	Yes				
Biochemistry^b^	Yes	Yes				
Toxicity (CTCAE v5)		Yes^c^				
ECG	Yes				Ind.	
Pulmonary function test	Yes				Ind.	
Bronchoscopy	Ind.^d^				Ind.^d^	
High resolution CT	Yes		Ind.^e^			
External ultrasound of the neck	Yes					
Written informed consent	Yes					
EGD with ≥4 bite-on-bite biopsies^f^	Yes^f^		Yes	Yes		
EUS with FNA^g^	Ind.			Yes		
PET-CT	Yes		Ind.^e^	Yes		Yes^h^
QoL questionnaires^i^	Yes			Yes		Yes^i^

*nCRT* neoadjuvant chemoradiotherapy, *CRE-1* first clinical response evaluation, four to six weeks after completion of nCRT, *CRE-2* second clinical response evaluation, 10–12 weeks after completion of nCRT, *Yes* test will be performed, *Ind* test will be performed only on indication, *ECOG* Eastern Cooperative Oncology Group, *CTCAE v5* Common Terminology Criteria for Adverse Events version 5, *ECG* Electrocardiography, *CT* computed tomography, *EGD* esophagogastroduodenoscopy, *EUS* endoscopic ultrasonography, *FNA* fine-needle aspiration, *PET-CT* positron emission tomography with computed tomography, *QoL* quality of life

^a^ Hematology: complete blood count and differential blood count;

^b^ Biochemistry: serum protein, albumin, sodium, potassium, chloride magnesium, serum creatinine, eGFR, bilirubin, alkaline phosphatase, AST, and pregnancy test if indicated;

^c^ Toxicity according to the CTCAE v5 will be assessed after each cycle of chemotherapy;

^d^ Bronchoscopy: in case of suspected tracheobronchial invasion based on other diagnostics;

^e^ In case of histological evidence of locoregional residual disease at CRE, a whole body PET-CT scan or CT scan will be made to exclude distant metastases;

^f^ Only during EGD at baseline it suffices if regular biopsies are taken instead of bite-on-bite biopsies;

^g^ FNA will be performed of all suspected lymph nodes based on prior PET-CT and based on assessment during EUS;

^h^ Follow-up PET-CT scans will be made at 16 and 30 months after completion of nCRT in patients who show a clinically complete response after CRE-2 to allow for comparison of distant dissemination rate in the future SINO trial;

^i^ QoL questionnaires EQ-5D, QLQ-C30, QLC-OG25 and Cancer Worry Scale will be taken at baseline, between CRE-2 and surgery. In patients who show a clinically complete response after CRE-2 and undergo immediate surgery questionnaires will also be taken at 6, 9, 12, 16, 20 and 24 months after completion of nCRT to allow for comparison in the future SINO trial

#### Neoadjuvant chemoradiotherapy

All patients will receive nCRT according to the CROSS regimen which consists of five weekly cycles of carboplatin intravenously (iv) at an area under the curve (AUC) of 2 mg/ml/min and paclitaxel iv at a dose of 50 mg/m2 on days 1, 8, 15, 22 and 29 with concurrent 41.4 Gy external beam radiotherapy given in 23 fractions of 1.8 Gy, 5 fractions per week, starting at the day of the first cycle of chemotherapy [4].

#### CRE-1

**Fig. 2:**
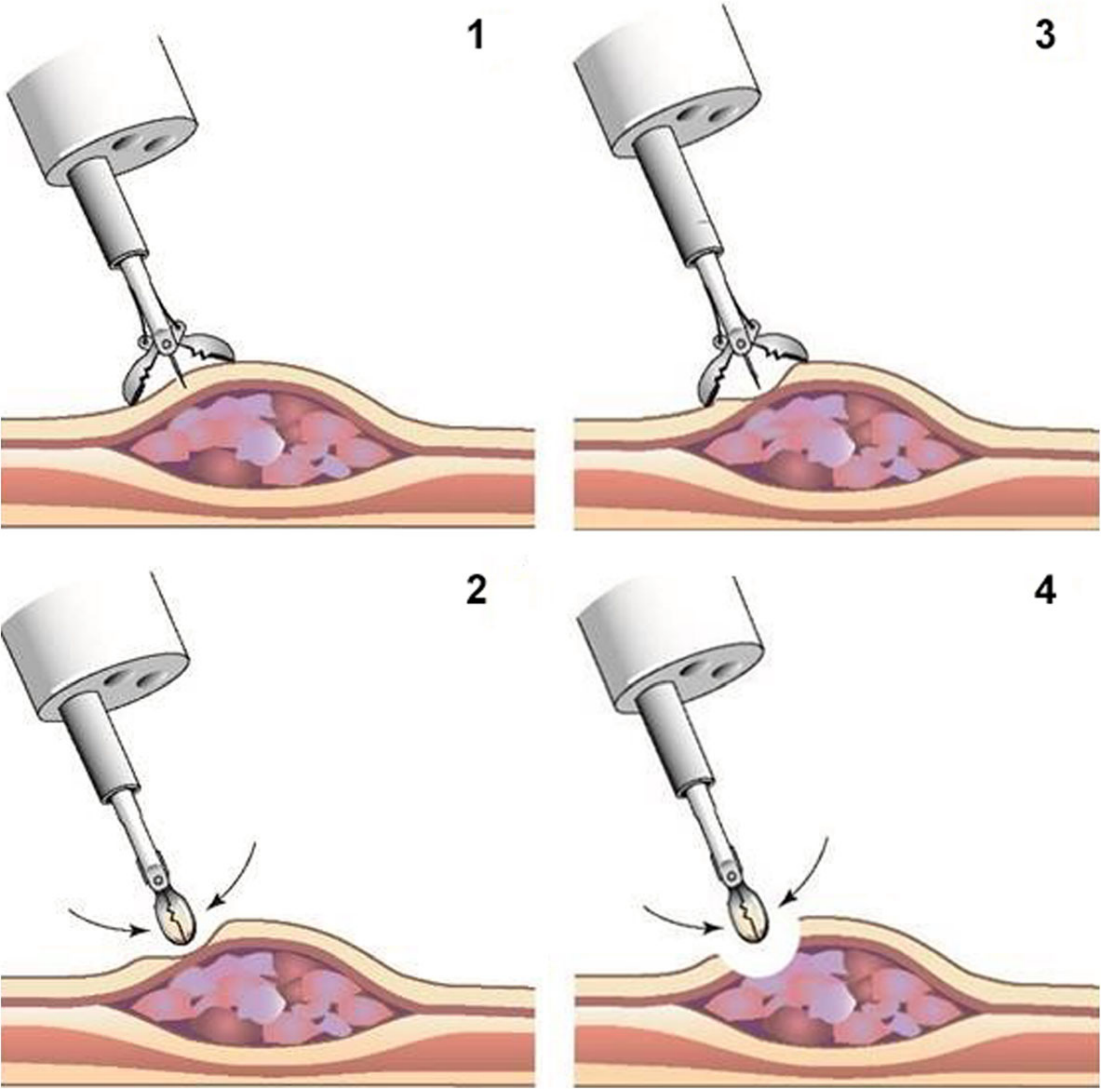
Bite-on-bite biopsy. During bite-on-bite biopsy [1–4] an extra biopsy [3, 4] is taken at the location of the previous biopsy [1, 2]. This procedure hypothetically increases the chance of detecting residual submucosal disease compared to conventional biopsies [1, 2]. (from: Noordman BJ, Wijnhoven BPL, Lagarde SM, Biermann K, van der Gaast A, Spaander MCW, et al. Active surveillance in clinically complete responders after neoadjuvant chemoradiotherapy for esophageal or junctional cancer. Dis Esophagus. 2017;30 [9]:1–8. With permission of Oxford University Press)

Patients will undergo a first clinical response evaluation (CRE-1) four to 6 weeks after completion of nCRT, consisting of EGD with at least four bite-on-bite biopsies (Fig. 2; Table 1). In every patient bite-on-bite biopsies will be taken within the area of the primary tumor. When performing bite-on-bite biopsies, a second biopsy is taken at the exact location of the first biopsy. In total at least four but preferably more bite-on-bite biopsies (≥ 8 biopsies) have to be collected. In case suspected lesions are present, bite-on-bite biopsies should also be taken of these lesions. If an ulcer is present, bite-on-bite biopsies should be taken at the border where normal mucosa meets ulcerative tissue. Photographs and/or a video will be taken for future reference.

In case of histological evidence of locoregional disease as well as in case of endoscopic non-traversable stenosis, CRE-1 will be considered positive and the patient will be identified as incomplete responder. These patients will receive an additional PET-CT scan to exclude distant metastases and will undergo subsequent esophagectomy. At this moment a CT scan could also suffice, because in fact patients with a positive CRE-1 will drop out of the trial. Patients with distant metastases will receive palliative care according to local protocol (Fig. 1). For patients without histological evidence of residual tumor, surgery will be postponed for another 6 weeks until the second clinical response evaluation (CRE-2) 10–12 weeks after completion of nCRT.

#### CRE-2

CRE-2 will be performed 10–12 weeks after completion of nCRT (Fig. 1). At this stage, patients without histological evidence of residual tumor at CRE-1 will undergo PET-CT scan, followed by EGD with bite-on-bite biopsies, and EUS with FNA of suspected lymph nodes (Table 1). PET-CT scans will be made prior to endoscopic response evaluations to allow for histological confirmation of PET positive intramural lesions and cytological confirmation of PET positive lymph nodes. A detailed PET-CT protocol has been written which will be used by all participating centers. In brief, all PET-CT scans will be made at 60 ± 5 min after injection of 2.96 MBq/kg 18F-FDG and will be performed according to the EANM guidelines version 2.0 [10]. Low-dose CT scans will be performed only for attenuation correction and anatomical correlation of PET images. Patients included in this study must have their CRE-2 PET-CT scans done on the same or identical type of scanner, under strictly the same conditions as their baseline PET-CT scan. PET-CT scans will be analyzed qualitatively. Lesions will be considered positive if any 18F-FDG uptake in the lesion is above the adjacent esophageal background uptake [11]. At least 4 bite-on-bite biopsies will be taken in the same way as during CRE-1. Subsequently, the entire esophagus will be assessed with EUS for presence of suspected lymph nodes. The two (or if possible three) most suspected lymph nodes will be sampled with FNA. EUS criteria to define a lymph node as suspected are lymph nodes that are round, hypoechoic and larger than 5 mm. However, based on experience of the preSANO and SANO trials it is known that about 50% of positive nodes after nCRT do not meet these endosonographic criteria. Therefore, in case of any doubt FNA should be performed and preferably lymph nodes are sampled even in case of low suspicion. Response evaluations are not for nodal staging only but for detection of any residual tumor regardless of the origin of the tumor cells. Contamination by malignant cells from the primary tumor will thus have the same clinical consequence as malignant cells from a positive lymph node. Therefore, FNA will also be performed if lymph nodes are located behind the (original) primary tumor. Photographs and/or a video will be taken for future reference.

Patients with distant metastases detected during CRE-2 will drop out of the study and receive palliative care (Fig. 1). Patients without distant metastases and without histological evidence of locoregional residual disease will be identified as clinically complete responders. In case of histological evidence of locoregional disease as well as in case of endoscopic non-traversable stenosis, CRE-2 will be considered positive and patients will be identified as incomplete responders. CRE-2 will not be considered positive if PET-CT suggests locoregional disease in the absence of histological confirmation. Both clinically complete and incomplete responders will undergo an esophagectomy shortly after CRE-2. If for any reason (e.g. poor general condition after nCRT) esophagectomy is postponed for more than 4 weeks after CRE-2, another CRE (CRE-2b) will be performed just prior to the planned operation.

#### Surgery

All patients without distant dissemination will undergo an open, hybrid or minimally invasive transthoracic esophagectomy (McKeown or Ivor Lewis) with at least two-field (chest and abdomen) lymphadenectomy. Selection of surgical technique will depend on patient and tumor characteristics as well as local expertise and preference.

#### Pathology

All biopsies will be evaluated by expert gastrointestinal pathologists. First, regular HE slides will be analyzed for vital tumor cells. If no malignancy can be identified, two or three additional sections will be performed and analyzed. In case the presence of vital tumor cells is still uncertain, extra diastase-periodic acid Schiff (dPAS) and (pan) keratin staining will be performed. Analysis of three additional sections and dPAS and keratin staining will always be performed if signet-ring cell carcinoma or a poorly cohesive carcinoma with mucin production was originally diagnosed. In case the pathological examination of the biopsy specimen concludes an uncertain outcome or high-grade/severe dysplasia, a second expert gastrointestinal pathologists will revise the specimen. In case of a discordant outcome, the specimens will be reviewed by a third independent expert pathologist. A final diagnosis will be made only if at least two pathologists agree. In case the consensus diagnosis concludes high-grade/severe dysplasia, the CRE will be considered positive. In case the outcome remains uncertain, the biopsies will also be considered positive for patients’ safety.

The resection specimen will be evaluated by two independent pathologists. Pathological examination will be performed according to standard protocol. Pathology reports should contain at least histological type, tumor size, proximal and distal resection margins, circumferential resection margin, invasion depth, grade of differentiation, lymphovascular invasion, number of resected nodes, number of resected positive nodes, tumor regression grade (TRG), ypTNM stage and prepTNM stage [12]. Microscopically radical resection (R0) will be defined as a tumor-free resection margin (margin > 1 mm not required). TRG will be categorized into four grades according to Chirieac: [9] TRG1: no residual carcinoma, characterized by microscopic evidence of radiation induced tissue injury, regenerative changes, and fibrosis extending through the layers of the esophageal wall. There is no histologically identifiable residual carcinoma. TRG2: 1–10% residual carcinoma, characterized by rare individual carcinoma cells present in fibrotic tissue at the primary site. TRG3: 11–50% residual carcinoma, characterized by microscopic foci of carcinoma cells present at the primary site. TRG4: greater than 50% residual carcinoma, characterized by substantial carcinoma remaining at the primary site. Pathological staging will be performed according to the 8th Edition of the AJCC TNM Classification for Esophageal Cancer [13]. The pathologist should try to identify at least 15 lymph nodes according to the National Comprehensive Cancer Network (NCCN) guidelines. However, the preferred average number of dissected and identified lymph nodes is more than 23 [14].

#### Follow-up

Patients having cCR in the preSINO trial can be included into the control arm (standard esophagectomy) of the planned future SINO trial as well. Since quality of life will be an important endpoint in the SINO trial, quality of life will be measured in patients included in the preSINO trial with EQ-5D, QLQ-C30, QLC-OG25 and Cancer Worry Scale questionnaires (Table 1) [15–18]. These questionnaires will be taken in all patients at baseline (pre-treatment) as well as between CRE-2 and surgery (3 months after completion of nCRT). In patients who show cCR after CRE-2 and undergo immediate surgical resection, the questionnaires will be taken at another six time points, i.e. 6, 9, 12, 16, 20 and 24 months after completion of nCRT.

A second important endpoint for the planned future SINO trial is the distant dissemination rate. In the experimental arm (active surveillance) of the SINO trial, PET-CTs will be frequently made during response evaluations. To compare the distant dissemination rate between both study arms, patients included in the control arm should undergo follow-up PET-CT scans as well, albeit less frequently. PET-CT scans will be made at 16 and 30 months after completion of nCRT in patients who show cCR after CRE-2 (Table 1). These patients must have their follow-up PET-CT scans done on the same or identical type of scanner, under strictly the same conditions as their baseline and CRE-2 PET-CT scan.

### Study endpoints

In line with the preSANO trial, it is hypothesized that for an active surveillance strategy in a future SINO trial TRG2 residual tumors (1–10% residual carcinoma) can be safely missed since the tumor will likely be detected in a resectable stage during a subsequent response evaluation. However, residual nodal disease without residual disease at the primary tumor site (ypT0N+) after nCRT tends to occur more frequently in patients with SCC than in patients with AC. Based on a large Dutch CROSS cohort comprising participants of the CROSS-I and CROSS-II trials, post-CROSS cohort and the preSANO trial, 7% of patients with SCC and 3% of patients with AC had ypT0N+ stage after nCRT according to CROSS. Therefore, the primary endpoint of the preSINO trial is the accuracy of both CRE-1 and CRE-2 combined for detecting TRG3–4 residual tumor (> 10% residual carcinoma) or TRG1–2 (≤10% residual carcinoma) with ypN+ (any residual nodal disease) in the surgical resection specimen. Secondary endpoints are the accuracy of both CRE-1 and CRE-2 combined for detecting pathologically non-complete response in both the primary tumor and regional lymph nodes (TRG2–4 or ypN+) as well as the association between the outcome of each single diagnostic modality and the pathological response (TRG, ypN) in the surgical resection specimen.

### Patient safety

Stop rules have been defined to guarantee maximum patient safety during the trial. A data safety and monitoring board (DSMB) will be established that will test the stop rules repeatedly. In case a stop rule is reached, the trial will be stopped immediately in all participating centers. Patients that have already been included and that have finished neoadjuvant treatment will not undergo any further research-related tests. These patients will be scheduled for surgical resection with minimal additional delay. Patients that are still receiving nCRT will continue their treatment and will undergo subsequent surgical resection according to standard protocol for esophageal cancer.

The following safety parameters will be closely monitored:

- The number of clinically relevant iatrogenic perforations resulting in an abscess, empyema and/or sepsis,
- The number of other treatment related complications (e.g. bleeding, aspiration, myocardial infarction) that lead to cancellation or postponement of surgery for at least 14 days.
- the number of patients that undergo a macroscopically or microscopically non-radical (R2 or R1) resection.

### Statistical analysis

#### Sample size calculation

In the preSANO trial, missing 10% of the patients with TRG3–4 residual tumor at the site of the primary tumor (false-negative rate of 10%) was considered acceptable in order to start an RCT comparing active surveillance with standard resection (SANO trial) [19]. In the preSINO trial, regional lymph nodes will also be evaluated. The acceptable false-negative rate will be increased with 2% because survival of patients with ypN+ is worse than patients who have ypN0, regardless of whether they are missed during clinical response evaluations or not. This increase of 2% will also prevent an exceedingly large sample size. Hence, missing 12% of the patients with TRG3–4 or TRG1–2 with ypN+ residual disease (false-negative rate of 12%) will be considered acceptable.

In the preSINO trial only patients with SCC will be included, while in the preSANO trial three quarters of patients had AC. Accounting for the better response to CROSS of patients with SCC than patients with AC (and thus a different distribution of TRG and ypN stages), a false-negative rate of 12% in a Dutch cohort with a majority of patients with AC corresponds to an allowed false-negative rate of 19.5% in a cohort of only patients with SCC.

With a power of 80%, a significance of 5% and an expected sensitivity of 89% for detecting TRG3–4 or TRG1–2 with ypN+ residual disease (from the preSANO trial), sample size calculations resulted in a required sample size of 133 patients with TRG3–4 or TRG1–2 with ypN+ residual disease to show that the sensitivity of the CREs is at least 80.5%.

Since 34% of patients with SCC showed TRG3–4 or TRG1–2 with ypN+ residual disease after nCRT according to CROSS, this results in a total sample size (TRG1–4 and any ypN stage) of 391 patients. To allow for a 15% drop-out (e.g. patients who do not undergo surgery due to metastases or due to patients’ choice to undergo an active surveillance strategy, ***460*** patients will be included. After interim analyses (performed at 50, 100, 200 inclusions) the distribution of TRG stages will be checked. According to the rate of TRG3–4 and TRG1–2 with ypN+ in these interim analyses, a new total sample size will be recalculated.

#### Data analysis

For primary data analysis the results of PET-CT, endoscopic bite-on-bite biopsies and EUS with FNA during CRE-1 and CRE-2 will be combined into one overall conclusion (cCR or clinically non-complete response). The number of true-positives, false-positives, true-negatives and false-negatives will be assessed. Based on these values, sensitivity, specificity, negative predictive value and positive predictive value will be calculated for CREs predicting TRG3–4 or TRG1–2 with ypN+ residual disease. Similarly, these values will be calculated for CREs predicting TRG2–4 or ypN+ residual disease (any locoregional residual disease). For secondary analysis, multiple imputation will be used for missing TRG scores of patients who have cCR after CRE-2 and who underwent subsequent active surveillance instead of surgical resection. Moreover, outcome of the single diagnostic modalities will be correlated with pathological response using χ^2^ test. An interim analysis will be performed after 50, 100 and 200 patients have been included. This analysis will be performed similarly to the primary data analysis. The number of true-positives, false-positives, true-negatives, false-negatives and the distribution of TRG stages together with the results of the assessment of both stop rules will be reported to the DSMB and IRB. If during these interim analyses the proportion of TRG3–4 and TRG1–2 with ypN+ patients is higher or lower than expected, the sample size will be recalculated to decrease or increase the number of patients that have to be included in the trial accordingly.

## Discussion

This preSINO study is a diagnostic trial, investigating the combination of diagnostic modalities that has been tested previously in the preSANO trial. It will provide evidence concerning the optimal diagnostic strategy for detecting residual locoregional disease after nCRT and, if successful, will be applied in a future SINO trial comparing active surveillance with standard esophagectomy in patients with esophageal SCC.

Esophageal SCC is a major public health issue for Central, East- and South-East Asian countries. Highest incidences are found in these areas with rates up to 13.6 per 100,000 in men and 4.3 in women (compared to a global average of 7.7 in men and 2.8 in women). In China, esophageal cancer is the fourth most common cancer, making it a major (financial) burden for Chinese national healthcare [20]. Hence, a potential reduction in the number of esophagectomies might not only have a positive impact on patients’ wellbeing, but also on the burden of esophageal cancer on Chinese healthcare systems.

Active surveillance after nCRT has been proposed for esophageal cancer since high pCR rates are being achieved by current nCRT regimens [5]. The Dutch CROSS trial showed that 5-year overall survival significantly increased from 33 to 47% by applying nCRT consisting of carboplatin and paclitaxel with concurrent 41.4 Gy radiotherapy prior to surgical resection [3]. Following this nCRT regimen, 49% of patients with SCC and 23% of patients with AC had pCR. However, only a small part of the included patients had SCC (23%) [4]. The Chinese NEOCRTEC 5010 trial showed that 3-year overall survival increased from 58.9 to 69.1% by giving nCRT consisting of vinorelbine and cisplatin with concurrent 40.0 Gy radiotherapy prior to surgical resection [21]. In this Chinese trial including only esophageal SCC, 43.2% of patients achieved pCR. Although pCR rates in patients with SCC are comparable between both regimens (49% vs. 43.2%), the CROSS regimen has been proposed as optimal nCRT regimen for the preSINO trial and future SINO trial. The major issue with nCRT according to the NEOCRTEC 5010 regimen is the relatively high toxicity rate. In the CROSS trial fewer grade 3–4 adverse hematological events (7.6%) occurred compared to 54.3% in the NEOCRTEC 5010 trial [4, 21].

The significance of the current preSINO study is that after nCRT according to CROSS, pCR rates tend to be higher in patients with SCC compared to patients with AC (49% vs 23%) [4]. In these patients surgical resection can potentially be postponed or even omitted. Hence, almost half of patients with SCC could benefit from an active surveillance strategy. However, the results of the preSANO trial cannot be simply extrapolated to the planned SINO trial since the preSANO trial included only 21 patients (17%) with SCC who underwent bite-on-bite biopsies and 43 patients with SCC overall (21%) [8].

Compared to the preSANO trial, the current study differs on several aspects. In the preSANO and subsequent SANO trial, operable patients who underwent or who were planned to undergo nCRT according to the CROSS regimen are/were considered eligible [7, 8]. However, patients with AJCC cN3 disease have a high risk of an incomplete locoregional response and a low chance of remaining tumor-free during follow-up in the planned future SINO trial. Also, these patients often have suspected lymph nodes outside the maximum tolerated radiation field and can therefore not receive CROSS chemoradiotherapy. Since inclusion of cN3 patients in the preSINO and SINO trial could reduce the proportion of cCR and pCR, patients with cN3 disease will not be included in the preSINO trial. Moreover, in the preSANO trial PET-CT was used to identify distant metastases at baseline and preoperatively. In the preSINO trial, patients who have a positive CRE-I (i.e. histologically proven locoregional residual disease or a non-traversable tumor) are also allowed to receive a high-dose CT scan instead of a PET-CT scan in order to identify distant metastases. Although high-dose CT scan is inferior to PET-CT scan in detecting distant metastases for esophageal cancer [22], Chinese insurance does not yet cover a preoperative PET-CT scan. However, both in the present preSINO trial and in the planned subsequent SINO trial the group of patients with a positive CRE-I (and CRE-II) will be excluded. Therefore, performing a high dose CT scan instead of PET-CT scan after a positive CRE-I will not affect the preSINO and SINO trials.

Furthermore, in the preSANO trial the reference standard that has been used for calculating the accuracy of the CREs was TRG in the resection specimen [8]. This grading system evaluates the response of the primary tumor site to neoadjuvant treatment only. However, the modalities used in the CREs also evaluate residual nodal disease. After nCRT, 4–9% of patients with SCC have a pathologically complete response at the primary tumor site with residual disease in the regional lymph nodes (ypT0N+), compared to 3–5% of patients with AC [23–25]. For these patients, higher ypN stage is correlated with worse overall survival [25]. However, these studies did not clearly specify the nCRT regimens that patients received. Moreover, the majority of patients probably did not receive nCRT according to the CROSS regimen since they were included before the results of the CROSS trial were published [23, 24]. Accordingly, data of a large Dutch CROSS cohort comprising patients that were included in the CROSS-I and CROSS-II trials, post-CROSS cohort and the preSANO trial were analyzed. In this large CROSS cohort, 8 of 122 patients with SCC (7%) and 11 of 415 patients with AC (3%) who underwent nCRT followed by a surgical resection had ypT0N+ stage. The percentage of patients with ypT0 who had ypN+ was comparable between SCC and AC (14% vs. 13%, resp.), suggesting that the higher rate of ypT0N+ patients is mainly caused by the better response of SCC to nCRT according to CROSS. In an active surveillance strategy that focusses on the primary tumor site only, patients who have ypT0N+ stage run a high risk of developing distant metastases. Therefore, in contrast to the preSANO trial, the preSINO trial will aim to identify patients with residual disease at the primary tumor site as well as in regional lymph nodes.

Similar to the preSANO trial, this trial will use bite-on-bite biopsies during endoscopic response evaluation. After nCRT for esophageal cancer, residual disease is found in deeper layers than the mucosa without residue in the mucosa itself in 24–40% of patients [26, 27]. In case of bite-on-bite biopsies, the second biopsy is taken at the exact same location as the first biopsy (Fig. 2). It has been suggested that this biopsy technique has the potential to reach into the submucosa, thereby increasing the rate of detecting residual tumor. The preSANO trial was started with regular endoscopic biopsies. However, sensitivity for detecting TRG3–4 residual tumor improved from 69 to 90% after the protocol had been changed from regular biopsies to bite-on-bite biopsies. Moreover, the preSANO trial demonstrated that performing FNA of suspected lymph nodes substantially improved detection of residual disease. It was shown that 29% of residual disease detected during CRE-2 alone was based on results of FNA only. Based on bite-on-bite biopsies only, sensitivity of both CRE-1 and CRE-2 combined for detecting TRG3–4 residual tumor was 83%. With the addition of FNA, this increased to 90% [8].

As demonstrated by the preSANO trial, the additional value of qualitative and quantitative PET-CT analysis in detecting early residual locoregional disease (up to 12 weeks after completion of nCRT) remains disputable. Qualitative PET-CT analysis alone yielded a reasonable sensitivity of 85% for detecting TRG3–4 residual tumor and a sensitivity of 80% for detecting TRG2–4 residual tumor. However, corresponding specificity was only 37%, caused by a high number of false-positives [8, 11]. After nCRT, radiation-induced esophagitis apparently increases 18F-FDG uptake throughout the irradiated esophagus including the primary tumor site, thereby increasing false-positive response evaluations. PET-CT detected novel distant metastases (i.e. not detected at baseline) in 9% of patients during CRE-2, saving them from unnecessary surgical resection [8, 11]. For this reason, in the current study PET-CT will only be used to detect distant metastases and will not be used for evaluating the primary tumor site. In the planned future SINO trial investigating an actual active surveillance strategy, PET-CT might become valuable as esophagitis will probably diminish over time, allowing detection of disease recurrence by qualitative and semi-quantitative analysis of small increments in 18F-FDG uptake.

Moreover, PET-CT will be used in follow-up to compare the distant dissemination rate between both arms of the future SINO trial. For patients undergoing active surveillance in the future SINO trial there is a hypothetical risk of increased distant dissemination since residual vital tumor might be missed during response evaluations. Current assumption holds that this process of spreading and seeding of tumor cells from the primary lesion is an early event. Hence, distant dissemination might have already happened at the time of diagnosis or during locoregional treatment [28]. This assumption is reflected by the substantial number of patients who develop hematogenous metastases after nCRT plus esophagectomy within 2 years after surgery [3, 4]. For this reason, PET-CTs will be performed until 2 years after surgical resection in the control arm (immediate surgery) of the planned future SINO trial. Since patients with a clinically complete response in the preSINO trial can be included in this arm of the SINO trial, these patients will also undergo follow-up PET-CT scans.

Although the preSANO trial showed that the combination of endoscopic bite-on-bite biopsies, EUS, and PET-CT was most accurate for detecting residual disease after nCRT, other measurements and modalities for response evaluation have been considered. To our knowledge, however, no other single modality or set of modalities has yet acquired an accuracy that equals the set from the preSANO trial. Moreover, because PET-CT, endoscopic biopsies and EUS are widely used for pretreatment clinical staging, physicians have experience with these techniques and they are available in most Chinese centers. One of the other measurements considered for the preSINO trial was the maximum tumor thickness on EUS as proposed by Jost et al. [29] In a cohort of 40 patients they found a sensitivity of 86% and specificity of 64% for detecting TRG 2–4 residual tumor. Based on these results, the preSANO trial investigated this method in 123 patients. In this larger cohort, a sensitivity of only 59% and specificity of 58% was found. Since these measurements did not add to the accuracy of the set of diagnostic modalities in the preSANO trial, it was decided that they are not included in the preSINO trial protocol [8]. MRI is another promising modality, showing sensitivities up to 97% for detecting residual tumor after nCRT [30, 31]. However, studies that find these high sensitivities all have poor corresponding specificities of at most 58%. In a future SINO trial, this overstaging of patients with a complete response to nCRT would result in unnecessary surgery. Blood-based biomarkers, including circulating tumor DNA, could also become useful for less invasive detection of residual disease. Methods have improved over the last few years, especially for predicting distant recurrences [32, 33]. To date, however, no biomarker has been identified that can accurately discriminate patients with incomplete locoregional response from patients with complete locoregional response.

If the preSINO trial shows that PET-CT, endoscopic bite-on-bite biopsies and EUS with FNA can detect TRG3–4 residual tumor in patients with esophageal SCC with a sensitivity of at least 81.5%, this combination of diagnostic tests will be used in a subsequent prospective trial comparing active surveillance with standard esophagectomy in patients with SCC and a clinically complete response after nCRT (SINO trial). Similar to the preSANO and SANO trials, it will be possible to include all patients of the preSINO trial having cCR into the control arm of the future SINO trial [7]. Of patients with SCC receiving nCRT according to CROSS, almost 50% achieve pCR and more than 50% is expected to have cCR. Consequently, the number of study subjects needed for the SINO trial can be substantially decreased compared to the SANO trial. Most probably, all patients needed for the control arm of the SINO trial will be derived from the preSINO trial.

## Data Availability

Not applicable.

## Abbreviations

18F-FDG: Fluorodeoxyglucose
AC: Adenocarcinoma
AJCC: American Joint Committee on Cancer
AUC: Area under the curve
cCR: Clinically complete response
CRE: Clinical response evaluation
CRE-1: First clinical response evaluation
CRE-2: Second clinical response evaluation
CROSS: Chemoradiotherapy for oesophageal cancer followed by surgery study
CT: Computed tomography
DSMB: Data safety and monitoring board
ECOG: Eastern Cooperative Oncology Group
EGD: Esophagogastroduodenoscopy
ESOSTRATE: Systematic surgery versus surveillance and rescue surgery in operable oesophageal cancer with a complete clinical response to radiochemotherapy
EUS: Endoscopic ultrasonography
FNA: Fine-needle aspiration
Gy: Gray
NCCN: National Comprehensive Cancer Network
nCRT: Neoadjuvant chemoradiotherapy
NEOCRTEC5010: Neoadjuvant chemoradiotherapy followed by surgery for squamous cell esophageal cancer
pCR: Pathologically complete response
PET-CT: Positron emission tomography with computed tomography
preSANO: Pre-surgery as needed for oesophageal cancer
preSINO: Pre-surgery if needed for oesophageal cancer
QoL: Quality of life
R1: Microscopically non-radical
RCT: Randomized controlled trial
SANO: Surgery as needed for oesophageal cancer
SCC: Squamous cell carcinoma
SINO: Surgery if needed for oesophageal cancer
TRG: Tumor regression grade
